# Bioelectrical impedance analysis—derived phase angle (PhA) in lung cancer patients: a systematic review

**DOI:** 10.1186/s12885-024-12378-4

**Published:** 2024-05-20

**Authors:** Melania Prete, Giada Ballarin, Giuseppe Porciello, Aniello Arianna, Assunta Luongo, Valentina Belli, Luca Scalfi, Egidio Celentano

**Affiliations:** 1 Division of Radiotherapy, Istituto Nazionale Tumori IRCCS Fondazione G. Pascale, Naples, 80131 Italy; 2 Department of Medical, Movement Sciences and Wellbeing, University of Naples “Parthenope”, Naples, 80133 Italy; 3 Epidemiology and Biostatistics Unit, Istituto Nazionale Tumori, IRCCS Fondazione G. Pascale, Naples, 80131 Italy; 4 Department of Public Health, Federico II University Hospital, Via Pansini 5, Naples, 80131 Italy; 5 Scientific Direction, Istituto Nazionale Tumori, IRCCS Fondazione G. Pascale, Naples, 80131 Italy

**Keywords:** Lung cancer, Phase angle, Body composition

## Abstract

**Background:**

Lung cancer is the second most diagnosed cancer in the world. Up to 84% of diagnosed patients have malnutrition, which can negatively affect quality of life and survival and may worsen with neoadjuvant treatment. Bioelectrical Impedance Analysis-Derived Phase Angle (PhA) in these patients could be a valid tool to assess the nutritional status in order to improve their condition.

**Methods:**

This review provides an update on PhA assessment in lung cancer patients over the past twenty years. We searched PubMed, Embase, Scopus, Web of Science, and Cochrane, for articles regarding the PhA obtained from Bioelectrical Impedance Analysis in lung cancer patients. The authors independently performed a literature search: sample size, patient population, study type, study dates, survival and interventions were evaluated. The final review included 11 studies from different countries.

**Results:**

Eight studies only considered patients with lung cancer, while three studies considered patients with different kind of cancer, including lung. Correlation data between PhA and age are conflicting. In patients undergoing clinical treatment and patients undergoing surgical treatment lower PhA was observed. A lower PhA is associated with a shorter survival. In three studies emerged a relationship between Karnofski Performance Status and Handgrip Strenght with PhA. From one study, univariate logistic regression analysis showed that higher PhA values represent a protective factor for sarcopenia.

**Conclusion:**

Our research underlined interesting, but not conclusive, results on this topic; however more researches are needed to understand the clinical meaning of PhA.

## Introduction

Lung Cancer (LC) is the second most diagnosed cancer worldwide, especially in males. Most recent data have shown an incidence of 2.2 million of new cases (11.4%) and 1,8 million of deaths (18.0%) occurred in 2020. It represents leading cause of cancer death in 93 countries [1]. Following diagnosis, 5-year survival rates ranges from 10 to 20% in most countries, with higher rates in Japan (33%), Israel (27%), and Korea (25%) [1]. In Italy, LC showed a 5-year survival of 16% in men and 23% in women [2]. LC aetiology is multifactorial and complex. In addition to a family history of LC, tobacco smoke currently represents the leading risk factor [2, 3]. Second-hand tobacco use may also increase LC risk, causing more than 3.000 deaths each year [4]. Other lung carcinogens include inhaled chemicals such as arsenic, cadmium and asbestos [5].

LCs are traditionally classified in small cell lung carcinoma (SCLC) and non-small cell lung carcinoma (NSCLC) divided into four major classes (adenocarcinoma, squamous cell carcinoma and large cell carcinoma) [6]. Conventional LC therapies include surgical intervention for resectable diseases and, in selected cases, a combination of radiotherapy (RT) and chemotherapy (CHT) for locally advanced or metastatic disease. Advancements in the understanding of LC molecular pathogenesis has led to the development of targeted strategies like immune checkpoint inhibitor (ICI) in first and later lines of treatment [7].

In addition to cancer-related symptoms, including chronic cough, dyspnoea, pain and adverse effects from anti-neoplastic treatments, LC patients’ may experience fatigue, weight loss or nutritional status alterations, such as malnutrition [8, 9]. Cancer patients are more likely to become malnourished, with a prevalence ranging from 20.0% to 80.0% [10]. Recent studies indicate that dietary nutrient deficiency in cancer patients may induce unintentional body weight loss to sarcopenia, up to cachexia [11]. It is known that malnutrition was prevalent in advanced LC patients [12]: up to 84% of them showed malnutrition status during illness, which can be worsened by ongoing neoadjuvant treatment [12–17]. This condition has been associated with poorer prognosis, decreased treatment response, poorer tolerance to treatment, lower quality of life (QoL) and increased healthcare costs [12, 18]. Additionally, sarcopenia, defined as progressive loss of muscle mass and functioning, is highly prevalent among LC patients ranging from 42.8% to 45.0%, in association with increased postoperative complications and increased risk of mortality, regardless of cancer stage and treatment [19]. Furthermore, LC is more commonly linked to cancer cachexia than other types of cancer [16], characterized mainly by a decrease in muscle strength, due to the loss of adipose tissue and skeletal mass [20, 21].

Body Composition (BC) is a crucial requirement for the overall body assessment of cancer patients: it can reflect the nutritional status of patients and predict clinical outcomes and prognosis [22]. Bioelectrical Impedance Analysis (BIA) is a simple, cost-effective and non-invasive method that measures electrical characteristics of human body, i.e. impedance (Z), through application of four electrodes and an applied alternate current, using single (SF-BIA) or multiple (MF-BIA) current frequencies. Z derives from resistive component (Resistance, R) and capacitive component (Reactance, X_c_), by equation Z = R^2^ + Xc^2^​. R indicates how much a substance opposes the flow of electric current: greater resistance indicates greater difficulty of passage. It can be affected by factors such as tissue density or hydration and cell membrane permeability. Reactance reflects the ease with which electricity can flow through tissues: high reactance means that there is more resistance of tissues and less conductivity. It can therefore indicate cellular and membrane integrity.

BIA-derived Phase Angle (PhA) is obtained as [arctangent (X_c_ /R) × 180°/π]. PhA represent an indicator of cellular health, cell membrane integrity, and better cell function: low values are indicators of apoptosis and cell matrix alteration [23, 24]. Therefore, BIA analysis and the use of the PhA have a good consistency in the application in cancer patients to evaluate nutritional and hydration status [25, 26]. Despite importance of nutritional status and BC for the clinical evaluation of cancer patients, these conditions remain in part an undertreated issue [27]. Thus, nutritional status assessment of these patients is essential for adequate nutritional support, and may also improve QoL and consequently survival post-diagnosis [28, 29]. In the literature, a large number of publications on BIA and in particular of PhA assessment, confirm its prognostic role in different types of cancer (e.g., breast, pancreatic and colon) [30–33]. Although PhA is a useful prognosis tool even in patients with LC [34], this has not been discussed in detail in scientific literature. So, our review aims to critically report and discuss available clinical data relating to PhA in LC patients, to provide a broad and clear picture of topic.

## Methods

The Preferred Reporting Items for Systematic Reviews and Meta-Analyses (PRISMA) guidelines were followed for performing the present review, considering the possibility of including both cross-sectional and longitudinal studies. Further details about PRISMA checklist and study protocol were provided in Supplementary File. Due to the study type, ethical approval was not required. This systematic revision is not currently registered in any database.

### Data sources

Authors independently performed a systematic literature research between 2000 and 2023 of the electronic databases PubMed, Embase, Scopus, Web of Science and Cochrane. The following terms were used as search strategy string on full texts: phase angle" AND (lung or pulmonary) AND (bioelectrical OR impedance OR bia) AND cancer”, "phase angle" AND (bia OR bioelectrical OR bioimpedance) AND cancer”. Zotero and EndNote X7 citation management software were used to manage citations.

### Criteria for analysis

In this review, cross-sectional, case–control, and longitudinal studies were included. The measurement of PhA values by BIA was a necessary and indispensable condition for an article to be included. The studies had to be present in the literature in English form and had to be published no earlier than 2000 to include the most recent evidence. Outcome of interest included associations between PhA and survival, mortality, or other variables related to LC patients. Furthermore, it was necessary that studies must have been conducted in the health field. Articles that did not meet these requirements were excluded from the review. All studies include a phase-sensitive BC measurement tool. In all selected studies PhA is calculated by the ratio of R to X_c_ equal to [arctangent (X_c_ /R) × 180°/π. BC parameters were not obtained by predictive regression models.

### Data extraction and analysis

To assess the suitability of the articles obtained from the literature search, authors carefully and meticulously examined all the titles and abstracts. Subsequently, the authors independently extracted the data from the papers and reported in an excel file. The data included: first author, year of publication, country of origin, design of study, sample size, age, sex, presence of control groups, type of tumour, methods used (BIA, BIVA), focusing on PhA.

## Results

### Selected studies

**Fig. 1:**
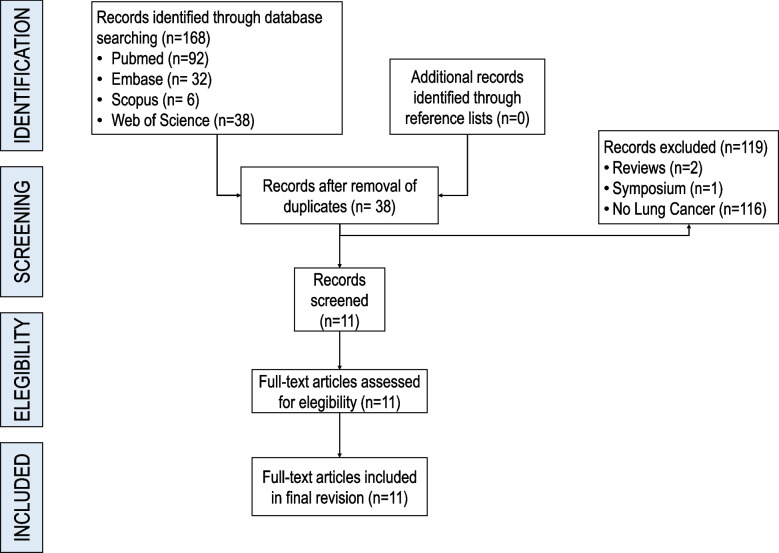
Flowchart

One hundred and sixty-five papers were identified from the systematic search, 92 from Pubmed, 32 from Embase, 6 from Scopus and 38 from Web of Science. After removing duplicates (*n* = 38), 120 articles were excluded, including 2 reviews, 1 symposium and 117 that did not concern LC. A total of 11 full-text articles were selected for eligibility, as shown in Fig. 1.

### General characteristics of included studies

This systematic review includes several types of studies: five prospective studies [35–39], three observational studies [40–42], two retrospective studies [34, 43] and one cross-sectional study [44]. Shi et al. showed the highest number of patients involved (804). The remaining selected scientific papers included 30 to 204 patients. Five studies included both males and females, six studies included males only [36, 38, 40–42, 44]. However, in two studies [36, 37], we do not know if the percentages of women and men present in the study referred to LC patients or patients with other kind of cancers. General characteristics of studies included in this review are shown completely in Table 1.

**Table 1:** General characteristics of included studies

**Study**	**Aim**	**Design**	**N**	**Sex**	**Age**^a^	**BIA Device**	**Electrode distribution**	**Vector**
Castanho et al. 2013 [44]	Assessing association between PhA and tumor volume in NSCLC patients	Cross-sectional	30	Male	65.6 (± 9.3)	BIA 450; Biodynamics, Seattle, Wash., USA	Whole body	No
Detopoulou et al. 2022 [40]	Investigate relationship between dietary habits and PhA in NSCLC patients	Observational	82	Male	65.8 (± 9.1)	Body stat Quad scan 4000	Whole body	No
Detopoulou et al. 2022 [41]	Assess differences in body composition and PhA in NSCLC patients	Observational	82	Male	65.8 (± 9.1)	Body stat Quad scan 4000	Whole body	No
Gupta et al. 2009 [34]	Investigate prognostic role of PhA in advanced NSCLC	Retrospective	165	Both	56.0 (± 9.1)	BIA-101Q: RJL Systems, Clinton Township, MI, USA	Whole body	No
Hui et al. 2014 [35]	Assessing association of PhA, hand grip strength and maximal inspiratory pressure with overall survival in patients with advanced cancer	Prospective	222	Both	55.0 (± 13.0)	RJL Systems Quantum IV (Clinton Township, Michigan)	Whole body	No
Ji et al. 2021 [36]	Explore association between sarcopenia and its confounding factors and evaluate PhA in the diagnosis of sarcopenia in older male patients with malignant tumors	Prospective	445	Male	Sarc 68.7 (± 3.6) No Sarc 70.7 (± 4.5)	Body composition analyzer InbodyS10 (Biospace Co., Seoul, Korea)	Whole body	No
Navigante et al. 2013 [37]	Determine relationship between weakness and BIA-derived PhA in a population of untreated cancer patients with fatigue	Prospective	41	Both	64.2 (± 10.6;)	Single-frequency BIA at 50 kHz	Whole body	No
Sánchez-Lara et al. 2012 [38]	Evaluate association between nutritional and inflammatory parameters with Health-Related Quality of Life (HRQoL) and prognosis in chemotherapy-naıve patients with advanced NSCLC	Prospective	119	Male	60.5 (± 12.5)	Body stat Quad scan 4000	Whole body	No
Shi et al. 2022 [39]	Assess association between PhA and overall survival in LC patients	Prospective	804	Both	60.60 (± 8.98)	Body composition analyzer: InBody S10, Biospace, Seoul, Korea	NS	No
Suzuki et al. 2023 [43]	To assess the predictive ability of the preoperative PhA for postoperative complications in patient undergoing surgery for primary LC	Retrospective	240	Both	68.6 (± 10.1)	Body composition analyzer: InBody 770, Tokio, Japan	NS	No
Toso et al. 2000 [42]	Compare measurement differences in patients with different LC stage (IIIB or IV) with those from healthy subjects; evaluate association between survival rate and impedance vector distribution	Observational	119	Male	Control 65.0 ± 8.0 Stage IIIB 67.0 ± 5.0 Stage IV 64.0 ± 7.0	BIA-101, RJL/Akern Systems, Clinton Township, MI, USA	Whole body	Yes

*PhA* Phase Angle, *NSCLC* non-small cell lung carcinoma, *LC* Lung Cancer, *Sarc* Sarcopenic

^a^age (standard deviation)

### Relationship between PhA, general characteristics and cancer features

Four studies investigated the potential relationship between age and PhA. In the study by Ji et al. [36], Pearson’s analysis showed an inverse correlation for age (*r* = -0.238 *p* =  < 0.001). Shi et al. [39], showed both for men and women, higher PhA in younger (for both, *p* < 0.001); Spearman’s correlation analysis also showed that PhA was significantly correlated with age (men, *r* = -0.46, *p* < 0.001; women, *r* = -0.24, *p* < 0.001). In Castanho et al. [44], Pearson’s correlation showed no significant results between PhA and age (*r* = -0.32). Suzuki et al. showed a negative correlation between age and PhA (*r* = -0.51; *p* < 0.001); Spearman’s correlation was used to assess the correlation between age and PhA.

Only 8 of 11, considered data on patients with LC, while 3 studies considered patients with different types of cancer, including LC. Three other studies investigate PhA in different types of cancer, including LC: they respectively include 8, 244 and 26 patients with LC [36, 37, 45]. All studies showed patients with confirmed cancer diagnosis: five studies evaluated NSCLC patients with stage III and IV [34, 38, 40–42]. Shi et al. included adenocarcinoma, squamous cell carcinoma, SCLC and other type of LC patients in different stage of disease (from I to IV) [39]. Suzuki et al. and Hui et al. showed data on LC patients but did not specify cancer type or stage [43, 45]. Cancer stage was not specified in two studies [36, 37]. Castanho et al. [44] considered LC patients presenting from stage IB to IIIB. Results showed, by multifactorial analysis of variance, correlations between PhA and tumour size (*r* = -0.55; *p* < 0.001) or Karnofski Performance Status (KPS) (*r* = 0.44; *P* < 0.05). Three studies included patients not undergoing CHT, RT and without specific treatment information [37, 39, 45] while newly diagnosed patients and/or patients with ongoing therapies were evaluated in remaining studies [34, 36, 38, 42]. Suzuki et al. [43] evaluated LC patients after surgery while patients were evaluated after cancer treatment in two studies [40, 41]. Castanho et al. [44] evaluated patients after surgery and after cancer treatment (CHT, RT).

### Relationship between PhA and body composition

The main associations identified in selected studies, between PhA and different variables, are shown in Table 2. In seven studies parameters related to BC have been evaluated [36, 39–41, 43, 44]. Castanho et al. [44] correlates PhA with arm circumference and weight loss over time. Authors also indicate that between patients undergoing surgery, those with lowest survival (52 days) showed a lower PhA (3.8°) and a High Extracellular Mass/Body Cell Mass (ECM:BCM) ratio (1:5), independent from tumour size. Whereas, in those treated medically, patients with a lower survival also had lowest PhA (3.9°) and highest ECM/BCM ratio (1:6), this were related to tumour volume (849 ml).

**Table 2:** Main associations between PhA and variables indicated in the studies

Study	Tumour characteristics	PhA vs Age and/or gender	PhA vs other variables	Statistical method
Castanho et al. 2013 [44]	LC stage IB to IIIB	age (*r* = -0.32)	Tumour size (*r* = -0.55; p < 0.001); KPS (r = 0.44; P < 0.05)	Pearson’s correlation
Detopoulou et al. 2022 [40]	NSCLC stage III and IV	NI	Lean mass (kg) (rho = 0.247, p = 0.02); dietary pattern rich in potatoes, meat and poultry (rho = 0.254, *p* = 0.02); MDS (rho = 0.251; *p* = 0.02)	Spearman’s correlation
Detopoulou et al. 2022 [41]	NSCLC stage III and IV	NI	Weight change (kg) (rho = − 0.01; *p* = 0.9); BMI (rho = − 0.02; *p* = 0.8); Waist Circumference Change (rho = − 0.08; *p* = 0.4); Lean Mass Change (rho = 0.201; *p* = 0.07)	Spearman’s correlation
Gupta et al. 2009 [34]	NSCLC stage III and IV	NI	Survival for PhA ≤ 5.3 (*r* = 7.6; *p* = 0.02); Survival for PhA > 5.3 (*r* = 12.4; *p* = 0.02); less survival for lower PhA (RR = 0.79; (95% CI: 0.64 to 0.97, *p* = 0.02)	Univariate Kaplan–Meier survival analysis Multivariate Cox proportional hazard model
Hui et al. 2014 [35]	LC stage not specified	NI	Survival (HR = 0.86, 95% CI: 0.74–0.99; *p* = 0 .04)	Multivariate Cox Regression analysis
Ji et al. 2021 [36]	NSCLC stage not specified	age (r = -0.238 p = < 0.001)	SMI (*r* = 0.463; p < 0.001); BMI (*r* = 0.450; p < 0.001); CC (*r* = 0.405; p < 0.001); HGS (*r* = 0.354; p < 0.001); sarcopenia (OR 0.309, 95% CI, 0.246 0.617; p < 0.001)	Pearson's correlations Univariate logistic regression analysis
Navigante et al. 2013 [37]	NSCLC stage not specified	NI	HGS (*p* = 0.007; 95% CI: 0.3843 to 0, 9717)	Mann–Whitney test
Sánchez-Lara et al. 2012 [38]	NSCLC stage III and IV	NI	Survival for PhA ≤ 5.8° (*p* = 0.032); OS (HR: 3.02; 95% CI, 1.2–7.11; *p* = 0.011)	Kaplan–Meier method Cox proportional hazards models
Shi et al. 2022 [39]	adenocarcinoma, squamous cell carcinoma, SCLC and other type of LC stage from I to IV	Men age (*r* = − 0.46, p < 0.001) Women age (*r* = − 0.24, p < 0.001)	NRS-2002 (men, *R* = − 0.25, p < 0.001; women, R = − 0.15, p < 0.001); survival (men p = 0.019 and women *p* = 0.121); mortality in men (HR = 0.79, 95% CI: 0.65–0.95, *p* = 0.015)	Logistic regression models Kaplan–Meier method
Suzuki et al. 2023 [43]	LC stage not specified	age (*r* = -0.51; p < 0.001)	BMI (rho = 0.29; *p* < 0.001); SMM (rho = 0.47; *p* < 0.001), Haemoglobin (rho = 0.33; *p* < 0.001), Albumin (rho = 0.33; *p* < 0.001), TLC (rho = 0.17; *p* < 0.001), PNI (rho = 0.32; *p* < 0.001)	Spearman’s correlation
Toso et al. 2000 [42]	NSCLC stage III and IV	NI	Survival for PhA ≤ 4.5° (*p* = 0.01); survival (OR = 1.25; 95% CI:1.01 to 1.55; *p* = 0.04)	Kaplan–Meier method Cox proportional hazards models

*PhA* Phase Angle, *LC* lung cancer, *KPS* Karnofski Performance Status, *NSCLC* non-small cell lung cancer, *SCLC* small cell lung cancer, *BIA* Bioelectrical impedance, *HGS* Handgrip-test, *NI* Not investigated, *OS* Overall Survival, *NRS-2002* Nutritional Risk Score-2002, *PNI* Prognostic Nutritional Index, *MDS* Mediterranean diet score, *TLC* total lymphocite count, *SMI* skeletal muscle mass index, *CC* calf circumference

Shi et al. [39] shows that male patients < 65 years, with a lower Body Mass Index (BMI) and lower cancer stage have higher PhA values, but the differences were not statistically significant. In Suzuki et al. [43], Spearman’s correlation showed a positive correlation between PhA and BMI (rho = 0.29; *p* < 0.001); no correlation with body Fat Mass (FM) was found. In Hui et al. [45] study, PhA was associated with several known prognostic variables, including Fat-Free Mass (FFM) and FFM index (FFMI). However, the Spearman correlation was weak (rho < 0.4; *p* < 0.001). Two articles assessed different aspect within the same LC patients' population (Stage IV, male patients), including PhA. In Detopoulou et al. [40] were found significant correlations between PhA and FFM (rho = 0.247; *p* = 0.02), but no significant correlation for waist and hip circumference (cm), waist-hip ratio, body fat (%), BCM (Kg), total body water (TBW), extra-cellular and intra-cellular water (ECW, ICW). In the other study, Spearman’s correlation shows no significance between PhA, anthropometric and BC variables [41]. In Wei Ji et al. [36], in addition to PhA, other variables were also examined, such as appendicular skeletal muscle mass (ASMM), BMI and skeletal muscle mass index (SMI). Pearson’s correlations showed a moderate correlation between PhA values and variables considered (ASMM *r* = 0.301, *p* < 0.001; BMI *r* = 0.450, *p* < 0.001; SMI *r* = 0.463, *p* < 0.001.

### Relationship between PhA, nutritional status and nutritional risk

PhA relationships with nutritional status and malnutrition screening scores were evaluated. Shi et al. indicated a significant correlation between PhA and some nutritional index: results of Spearman’s rank correlation test showed correlation with Nutritional Risk Score-2002 (NRS-2002) (men, *r* =  − 0.25, *p* < 0.001; women, *r* =  − 0.15, *p* < 0.001). No correlation between PhA and Patient-Generated Subjective Global Assessment (PG-SGA) score was found. Then, logistic regression analysis showed significant correlation between PhA, NRS-2002 score (men, *p* < 0.001; women, *p* < 0.001) and PG-SGA score (men, *p* < 0.001; women, *p* < 0.001) in both men and women, indicating an association with secondary clinical outcomes such as nutrition and well-being [39]. In Suzuki et al. [43], Spearman’s correlation showed a positive correlation between PhA and albumin (rho = 0.33; *p* < 0.001), considered a useful biochemical markers for nutrition assessment, and Prognostic Nutritional Index (PNI) (rho = 0.32; *p* < 0.001), a simple index obtained from serum albumin concentration and total peripheral blood lymphocyte count, used to assess the immune-nutritional status of patients who undergo surgery. In Hui et al. [45] study, PhA was associated with several known prognostic variables, including serum albumin, but correlation was weak (γ < 0.4, *p* < 0.001; Spearman correlation test). Detopoulou et al. [40] found significant correlations between PhA and dietary pattern (Food pattern 2) rich in potatoes, meat and poultry (rho = 0.254, *p* = 0.02). No significant results with PhA and other dietary patterns (food pattern 1: whole grains, fruits, vegetables; food pattern 3: high olive oil, low alcohol; food pattern 4: legumes, fish). Finally, in the same patient’s sample, Mediterranean Diet Score (MedDiet Score) was positively related to PhA changes (rho = 0.251; *p* = 0.02).

### Relationship between PhA, prognostic indices, quality of life and survival

Some of the selected studies evaluated the correlation between PhA and some prognostic indices, QoL scores and survival in patients with LC. Five out of eleven studies indicate patient survival data in relation to the PhA [34, 35, 38, 39, 42]; in two studies, indicators associated with survival were evaluated [34, 35, 38, 39, 42–44]. Sanchez-Lara et al. and Shi et al. have evaluated QoL in relation to PhA.

Multifactorial analysis of variance showed correlations between PhA and KPS (*r* = 0.44; *P* < 0.05) in cross-sectional study by Castanho et al. [44].

In Hui et al. [45] study, PhA was associated with several known prognostic variables, including the Palliative Performance Scale (γ = 0.18; *p* = 0.007), KPS (γ = 0.18; *p* = 0.007), Palliative Prognostic Score (γ = -0.21; *p* = 0.002), and Palliative Prognostic Index (γ = -0.22; *p* = 0.001). Unadjusted PhA (*P* = 0.001) was found to be significantly associated with overall survival, as indicated by Kaplan-Meyer curves analysis: a lower value is associated with poor survival (PhA < 3°, median 35 days; 95% CI, 29–41 days).

Sanchez-Lara et al. [38] evaluated the association of PhA, QoL’s dimensions EORTC QLQ C30 (QLQ-C30) and survival in patients with advanced NSCLC. No significant association between PhA and QoL scores were found. The bivariate survival analysis shows that PhA ≤ 5.8° was significantly associated with low overall survival; multivariate analysis indicate for highest PhA values a higher survival rate (HR = 3.02; 95% CI, 1.2–7.11; *p* = 0.011).

The results of Spearman’s rank correlation test in Shi et al. [39] showed correlation between PhA and QoL. It was found a L-shaped association between PhA and LC survival in both sexes (men *p* = 0.019 and women *p* = 0.121); an association between higher PhA and better survival resulted for men and women (*p* = 0.007 and *p* < 0.001, respectively). Kaplan–Meier survival curves for patients with high and low PhA values in different cancer stages showed longer OS in patients with high PhA than patients with low PhA, without taking account stage. Univariate Cox regression analysis showed that continuous PhA was significantly associated with mortality in men with LC (*p* = 0.015); also in women, PhA was significantly associated with survival (*p* = 0.029). After adjusting for several covariates, in a multivariate-adjusted Cox regression analysis PhA was identified as an independent risk factor for mortality in men (HR = 0.79, 95% CI = 0.65–0.95, *p* = 0.015), but not in women (*p* = 0.105) [39].

In Gupta et al., univariate Kaplan–Meier survival analysis showed statistically significant differences (*p* = 0.02) between patients with PhA <  = 5.3 (median survival = 7.6 months; 95% CI: 4.7 to 9.5; *n* = 81) and those with > 5.3 (12.4 months; 95% CI: 10.5 to 18.7; *n* = 84) [34].

No correlation with Charlson Comorbidity Index was found in Suzuki et al. (rho = -0.09; *p* = 0.16). Also, in this study, multivariate logistic analysis reveals that PhA (OR = 0.51, 95% CI: 0.29–0.90, *p* = 0.018) was an independent predictor of Clavien-Dindo grade ≥ II, index used for surgical complications [43].

Data from univariate survival analysis of Toso et al., stratified by the cancer stage, indicated that LC patients with a PhA ≤ 4.5° had significantly shorter survival compared to those who have a higher PhA (*p* = 0.01) (median of 3.7 vs 12.1 months in patients with a PhA ≤ 4.5° vs > 4.5°, respectively, from stage IIIB, and 1.4 vs 5.0 months in in patients with a PhA ≤ 4.5° vs > 4.5, respectively from stage IV) [42].

### Relationship between PhA, muscle strength and physical efficiency

Navigante et al. [37] evaluated weakness assessed with Hand-grip strength (HGS). In patients with LC only statistically significant result was linear correlation between grip work and PhA (*p* = 0.007), which was considered very significant (95% CI: 0.3843 to 0.9717).

In Hui et al., PhA was also associated with HGS, but correlation was not very strong (Spearman’s correlation γ < 0.4; *p* > 0.001) [35].

Wei Ji et al. have evaluated muscle strength and ASMM to define sarcopenia diagnosis: PhA had strongest correlation with SMI (*r* = 0.463) and HGS (*r* = 0.354). Logistic regression analysis adjusted for potential confounders showed that higher PhA values represent a protective factor for sarcopenia (OR 0.309, 95% CI, 0.246 0.617; *p* < 0.001) [36].

### Comparison between different groups and PhA

In the study of Toso et al. were reported differences between healthy subjects, patients with IIIB and IV stages.

Comparing patients with healthy controls was found a reduction in PhA value (resulting in a reduction in capacity, but not R. No significant differences between two groups of patients with IIIB and IV stages were found. However, a significant difference between patients with different stages (statistically lower in patients with higher stages) was observed for survival [42].

In Navigante et al. was carried out a comparison between different groups (healthy volunteers vs patients), but in reference to muscle strength (maximal muscle strength, mean muscle strength, median muscle strength) and not to PhA [37].

In the Hui et al. patients’ cohort (*n* = 204) with different types of cancer (including breast, gastrointestinal, head and neck and gynaecological) two different groups have been distinguished: patients with edema and without edema. Univariate analysis showed a reduced survival for PhA ≤ 3° vs PhA > 3° for total patients (*p* = 0.045) and no edema patients (*p* < 0.001). PhA ≤ 3° was associated with shorter survival in the non-oedematous cohort (HR 4.42, 95% CI 2.09–9.36, *p* < 0.001), but this association did not occur in the whole cohort (HR 1.44, 95% CI 0.99–2.09, *p* = 0.054) and in the cohort with edema (HR 1.04, 95% CI 0.67–1, 62, *p* = 0.85) (Cox regression analysis) [35].

Wei Ji et al. evaluated the association between PhA in older male patients with different types of malignancies, with sarcopenia (22.0%) and without. PhA in patients without sarcopenia was 5.02° (SD ± 0.72°), while in sarcopenic was 4.18° (SD ± 0.85°); this difference was statistically significative (*p* < 0.001) [36].

## Discussion

The present review aims to investigate the current data regarding PhA in LC patients. We did not find a large number of studies focused on the assessment of PhA, which made it difficult to reach a comprehensive conclusion on this topic. We found 11 studies evaluating the PhA value obtained from BIA in patients with LC. In order to choose the right cancer treatment and plan carefully, survival prediction is crucial. In any case, new tools are necessary to be applied in daily clinical practice.

In the latest years, a growing body of studies have evaluated the prognostic role of PhA not only in patients with LC, but also in patients with respiratory disease. Indeed, De Benedetto et al. reported that lower PhA is associated with a decreased muscle mass, muscle strength and exercise capacity in patients with idiopathic pulmonary fibrosis, regardless of body weight. Moreover, patients with Chronic Obstructive Pulmonary Disease (COPD) and lower PhA have reduced cell mass, evident skeletal muscle depletion, worsening gas exchange and an increased risk of all-cause mortality [46]. Similarly, a lower PhA has been associated with an increased risk of malnutrition, sarcopenia, fluid retention, systemic inflammation, symptoms, and poorer QoLin patients with cancer. Moreover, a lower PhA may be a novel prognostic factor of poorer overall survival and higher risk of postoperative complications in cancer patients [47].

Overall, lower PhA levels have been associated with poorer physical condition and shorter survival in patients with LC: in Sanchez-Lara et al. [38], Gupta et al. [30–32, 34, 48] and Toso et al. [42] LC patients with lower PhA had a shorter survival than patients with higher PhA. In addition, in Shi et al. [39] patients with a higher PhA had a better survival and PhA was an independent risk factor for mortality in men with LC. Navigante et al. [37] in cancer patients without edema, PhA values ≤ 3° were associated with mortality within three days of BIA analysis, while in sarcopenic patients the PhA value was reduced compared to non-sarcopenic patients.

Patients with a lower PhA also had a higher risk of complications after surgical procedures [36]. In the prospective observational study of Uccella et al., it was observed that PhA was an independent prognostic factor of optimal cytoreduction and postoperative complications among patients with primary diagnosis of advanced ovarian cancer [49]. Similarly, in the prospective observational study of Inci et al. (The Risk-Gin trial) the authors observed that patients undergoing surgery for gynecological cancer with PhA < 4.75° and HGS < 44 kg in both hands had a three-fold increased risk of 30 days severe postoperative complications [50].

Thus, in addition to being a marker of cellular function, muscle mass and nutritional status, PhA may be a predictive factor of acute catastrophic complications risk. Interestingly, PhA was weakly but significantly associated with other prognostic variables, suggesting that it captures some additional information compared to existing prognostic factors. Further studies are needed to examine physiological and cellular changes associated with PhA.

Gupta et al. have evaluated role of PhA in the prognosis of 52 patients with advanced colorectal cancer: patients with PhA ≤ 5.7º had an 8-months average survival rate (Kaplan–Meier method), while those with PhA > 5.57º had a 40-month average survival rate [32]. Bosy-Westphal et al. [51] showed that patients with PhA < the fifth percentile had a deterioration in nutritional and functional status, decreased QoL and increased morbidity and mortality. The fifth percentile was a clinically relevant indicator of cancer cachexia. In this context, Hui et al. [35]investigated the association between PhA and survival in individuals with terminal cancer, where the increment of 1 degree in PhA was associated with higher survival rates.

In the prospective observational study by Paiva et al. PhA is reported as an independent prognostic factor, and Norman et al. [52]showed that in cancer patients, PhA values (stratified by age, sex, and BMI) below the fifth percentile of reference corresponded to a significant deterioration in nutritional status. In addition, these patients showed decreased HGS, increased incidence of weight loss, dyspnoea, fatigue, and increased risk of mortality. In three articles positive correlation has emerged between HGS and PhA, so weakness is related to the reduction in PhA in cancer patients. In other patient’s populations, it was shown that impaired muscle strength was associated with a poorer prognosis [53, 54]. In addition, correlations between HGS, PhA and other BIA variables have emerged in adolescents and young adults [55].

In Navigante et al. [37], univariate logistic regression analysis showed that higher PhA values represent a protective factor for sarcopenia. In Pérez-Camargo et al. [56] palliative care patients with cancer (the most frequently were gastric cancer, gynecological cancer, LC, and haematological malignancies) and severe sarcopenia had a lower mean PhA (3.9°) compared to patients without sarcopenia (mean PhA was 4.1°) showing that PhA is an independent measurement that can be associated to detect sarcopenia. Moreover, authors found that sarcopenic patients had a shorter overall survival and an increased risk of death compared to patients without sarcopenia. A recent review [57] indicated that PhA and sarcopenia are related to LC prognosis through different mechanisms including inflammation and oxidative stress. Detection of sarcopenia and the evaluation of BC and PhA can be a valuable tool for identifying and timely intervention of the state of malnutrition of the cancer patient. Timely analysis of patient’s nutritional status is essential, as it allows to avoid significant loss of cell mass and lean mass, but ensures a proper nutritional approach in order to avoid aggravation of the general condition of the patient. Moreover, to obtain an accurate clinical interpretation of PhA, simultaneous assessment of hydration and BCM status is required [58]. These informations could be derived from the analysis of vector length on the R/Xc plot using Bioelectrical Impedance Vectorial Analysis (BIVA) (a scatterplot that represents R in X-axis and Xc in Y-axis divided by height in meters). In healthy subjects, a balance is observed between BCM and hydration status of FFM: malnutrition, sarcopenia and cachexia can lead to a loss of BCM and cell membrane surface area, provoking cell damage and a reduction in PhA [58].

According to the American Cancer Society (ACS), one of the first hallmarks of several types of cancer, including LC, is unexplained weight loss. In addition, treatments such as RT and CHT could generate side effects that cause inappetence to patients (e.g., mouth ulcers, nausea, vomiting). Tumour growth leading to extreme loss of appetite and weight in association with systemic signs of inflammation may be breeding grounds for cachexia [59]. Cachexia leads to a considerable loss of skeletal muscle mass (SMM) that cannot be completely compensated with traditional nutritional supports. Furthermore, it may be an underlying condition in patients with sarcopenia. We know that malnutrition and cachexia are often present in cancer patients [13]: this state can worsen the effects of anticancer therapies, with premature discontinuation of treatment, patient's QoL decreased and higher risk of mortality [60, 61]. Nutritional status in cancer patients, especially the elderly, should be evaluated before and during CHT [61]. However, evaluation and detection of malnutrition status is not simple and instantaneous in patients. Therefore, it is preferable and useful to use the different variables defined by the BIA, to evaluate the changes in cellular membrane and body water levels. Future research on the use of PhA in clinical practice will be valuable in establishing cut-off values to better categorize oncology patients [58].

### Limitations

To our knowledge, this is the most recent review focusing on the assessment of BIA-derived PhA in LC patients, however, we had to consider several limitations. Although several search engines outside PubMed were used, the review included only 11 studies of which only 8 were aimed at analysing PhA exclusively on patients with LC. Only four studies related to this topic were published in 2022, the other articles we included in our work were published between 2000 and 2013, so we do not have a large number of recent evidence.

Finally, accurate data on PhA relationships and other variables such as anthropometric values, HGS test, ECM/BCM ratio, tumour stage, tumour volume, etc., are absolutely necessary for a broader understanding of how PhA can be beneficial for this population. No information about PhA timing measurement by bioimpedance were provided in selected studies Monitoring the health of these patients is very important in order to be able to act promptly with targeted integrated therapies involving nutrition and physical activity, so further studies are needed. Patients with LC are at greater risk of malnutrition, sarcopenia and cachexia, with implications for physical function and overall QoL. Therefore, before making an assessment of the BC and PhA, it would be useful to consider the stage of the disease, cancer therapies carried out and the side effects, comorbidities and possible states of inflammation. All measurement should be performed in standard conditions and, in these specific cases, in days that do not correspond with cancer therapy. Patients with chronic respiratory disease could experience dysfunction in skeletal muscle mass (SMM) and BC changes as consequence of smoking, alcohol abuse, systemic inflammation, systemic oxidative stress, hormonal deficiencies, comorbidities, aging, and inappropriate diet [46, 47]. Moreover, fat-free mass depletion and decreased muscle strength are common features of these patients. In this context, usefulness of PhA as a health status marker was investigated by a growing body of study. Similarly, patients with cancer patients have multiple comorbidities (chronic kidney disease) which may impact BC and cellular function. Moreover, patients with cancer frequently have fluid retention including edema, ascites, and pleural effusions. These factors may complicate the interpretation of PhA because this parameter is affected by altered ECW/ICW, or fluid disruption. Therefore, given the few studies currently available and the high number of factors that can affect BC measurements (hydration status, concomitant intake of food, alcohol use, physical activity, menstrual cycle, use of specific drugs that increase cell retention and catabolism, etc.) it should maintain accurate standardization in measurement.

## Conclusions

The evaluation of PhA by BIA analysis in LC patients is not widely discussed in scientific literature. However, early identification in nutritional status changes in cancer patients represents a crucial aspect to improve patient’s quality of life both post-diagnosis and during and after anticancer therapies, avoiding possible states of malnutrition and sarcopenia, which can aggravate patient’s status. This systematic review shows an association between a very low PhA and an increased risk of a deficent physical condition, linked to reduced survival in lung cancer patients. In the selected studies, various cut-offs point for PhA have been reported that need to be interpreted with caution: to date, it is not possible to define a single threshold or cut off point for PhA due to technical differences in commercial BIA devices (single-, multiple-frequency and BIS). Given the high incidence of this cancer and the low number of studies on this issue, it would be important and necessary to make greater use of this screening method in clinical practice.

## Data Availability

All the data analysed for this review are present in the tables; there are no additional files.

## Abbreviations

ACS: American Cancer Society
ASMM: Appendicular Skeletal Muscle Mass
BC: Body composition
BCM: Body Cellular Mass
BIA: Bioelectrical Impedance Analysis
BIVA: Bioelectrical Impedance Vectorial Analysis
BMI: Body Mass Index
CC: Calf Circumference
CHT: Chemotherapy
COPD: Chronic Obstructive Pulmonary Disease
ECM: Extra-cellular Mass
ECW: Extra-cellular Water
FFM: Fat-Free Mass
FFMI: Fat-Free Mass index
FM: Fat Mass
HGS: Hand-grip strength
HRQoL: Health Related Quality of Life
ICI: Immune checkpoint inhibitor
ICW: Intra-Cellular Water
KPS: Karnofski Performance Status
LC: Lung Cancer
MDS: Mediterranean Dietary Score
MF-BIA: Multiple-frequency Bioelectrical Impedance Analysis
NRS-2002: Nutritional Risk Score-2002
NSCLC: Non-small cell lung carcinoma
OS: Overall Survival
PhA: Phase Angle
PG-SGA: Patient-Generated Subjective Global Assessment
PNI: Prognostic Nutritional Index
PRISMA: Preferred Reporting Items for Systematic Reviews and Meta-Analyses
QLQ – C30: Quality of Life Questionnaire – Cancer 30
QoL: Quality of Life
R: Resistance
RT: Radiotherapy
SCLC: Small cell lung carcinoma
SF-BIA: Single-frequency Bioelectrical Impedance Analysis
SMI: Skeletal Muscle Mass Index
SMM: Skeletal Muscle Mass
TLC: Total Lymphocyte Count
TBW: Total Body Water
Xc: Reactance
Z: Impedance
